# Supplementary material for: Observations of the seiche that shook the world
Source: Nat Commun. 2025 Jun 3;16:4777. doi: 10.1038/s41467-025-59851-7 (PMC12134380; doi:10.1038/s41467-025-59851-7)
Supplement: Supplementary file 1 — Supplementary Information [file 41467_2025_59851_MOESM1_ESM.pdf]

Supporting information for Observations of the seiche that shook the world

Thomas Monahan[1*], Tianning Tang[1,2†], Stephen Roberts[1†], Thomas A. A. Adcock[1†]

[1*]Department of Engineering Science, University of Oxford, Oxford, OX1 3PJ , United Kingdom.

[2*]Department of Mechanical and Aerospace Engineering, University of Manchester, Manchester, M13 9PL , United Kingdom.

*Corresponding author(s). E-mail(s): thomas.monahan@eng.ox.ac.uk;
†These authors contributed equally to this work.

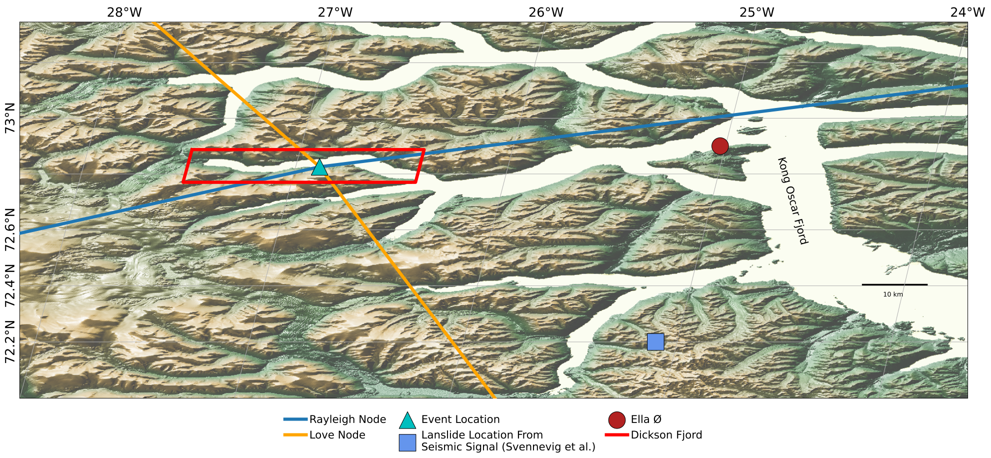

Supplementary Figure 1 Overview map of the study region including relevant adjacent locations. Background DEM is the Copernicus Global Digital Elevation Model [1]. Made with Cartopy Natural Earth [2].

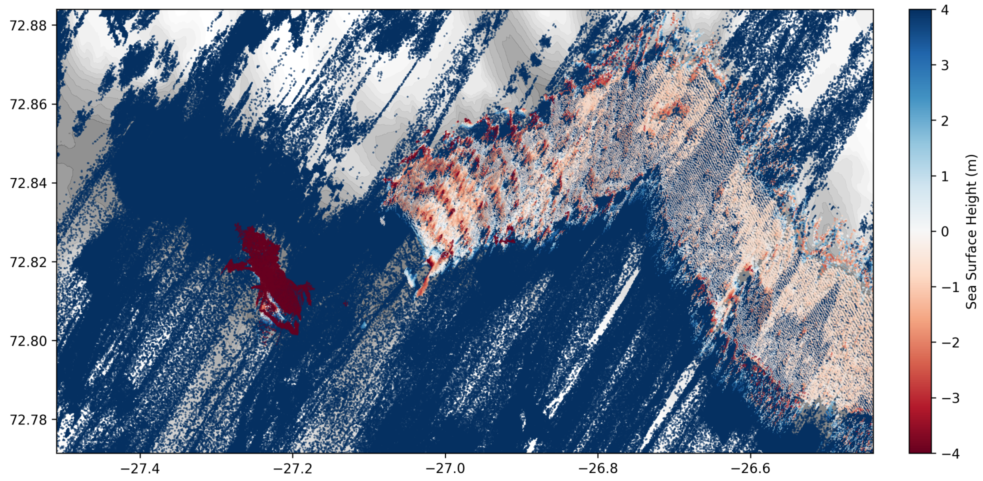

Supplementary Figure 2 Example SWOT observation with noise contamination. Data is processed using the same procedure described in Section 4.0.1. Observation produced by data file: SWOT_L2_HR_PIXC_005_292_021R_20231023T020832_20231023T020843_PGC0_01.

Supplementary Table 1
Bounding box for SWOT querying

Corner	Lat	Lon
Bottom Left	72.77	-27.51
Top Left	72.89	-27.51
Bottom Right	72.77	-26.42
Top Right	72.89	-26.42

Supplementary Table 2 Tidal Constituents

Constituent	Origin	Type	Period (hours)	CTD Amplitude (cm)
M2	Principal lunar	semidiurnal	12.42	43.19
S2	Principal solar	semidiurnal	12.00	23.00
N2	Elliptical lunar	semidiurnal	12.66	9.41
K1	Declinational luni-solar	diurnal	23.93	8.06
O1	Principal lunar	diurnal	25.82	7.49

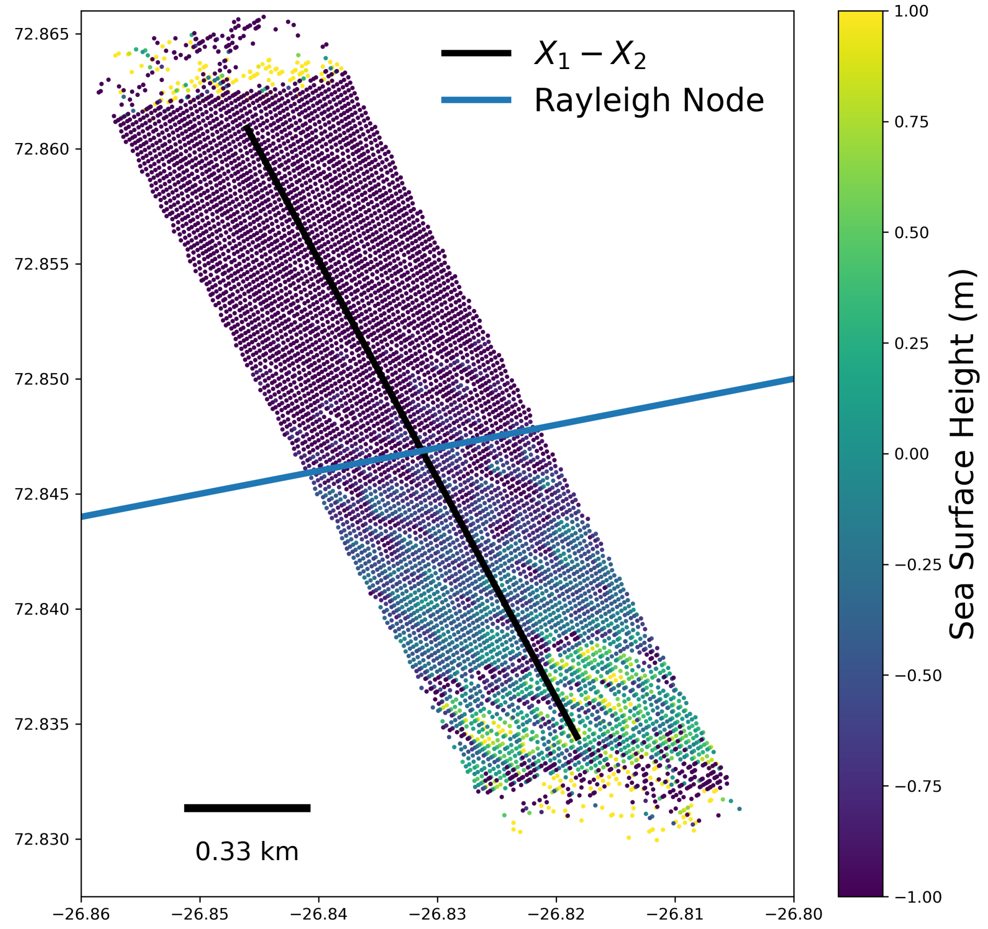

Supplementary Figure 3 Example SWOT cross-section used to compute the cross-channel slope. SWOT observations are colored according to sea-surface height.

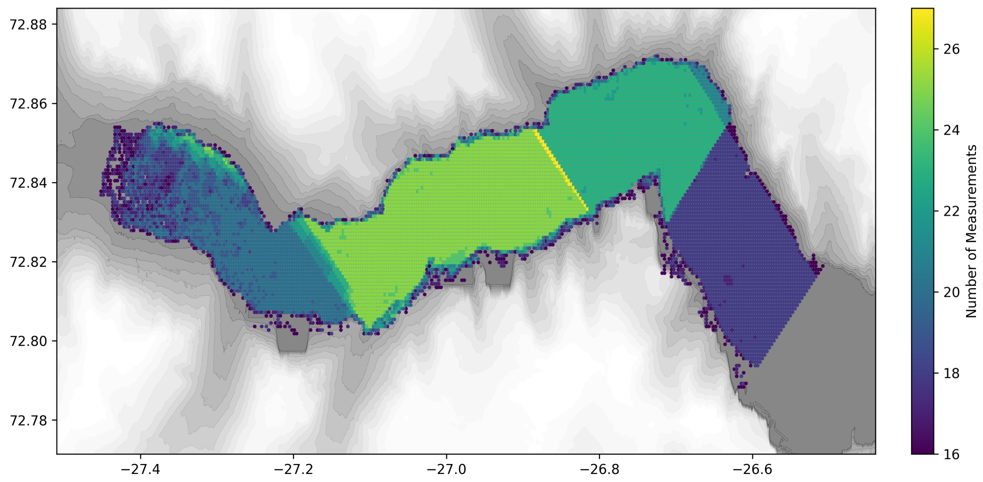

Supplementary Figure 4 Number of SWOT measurements available for tidal estimation.

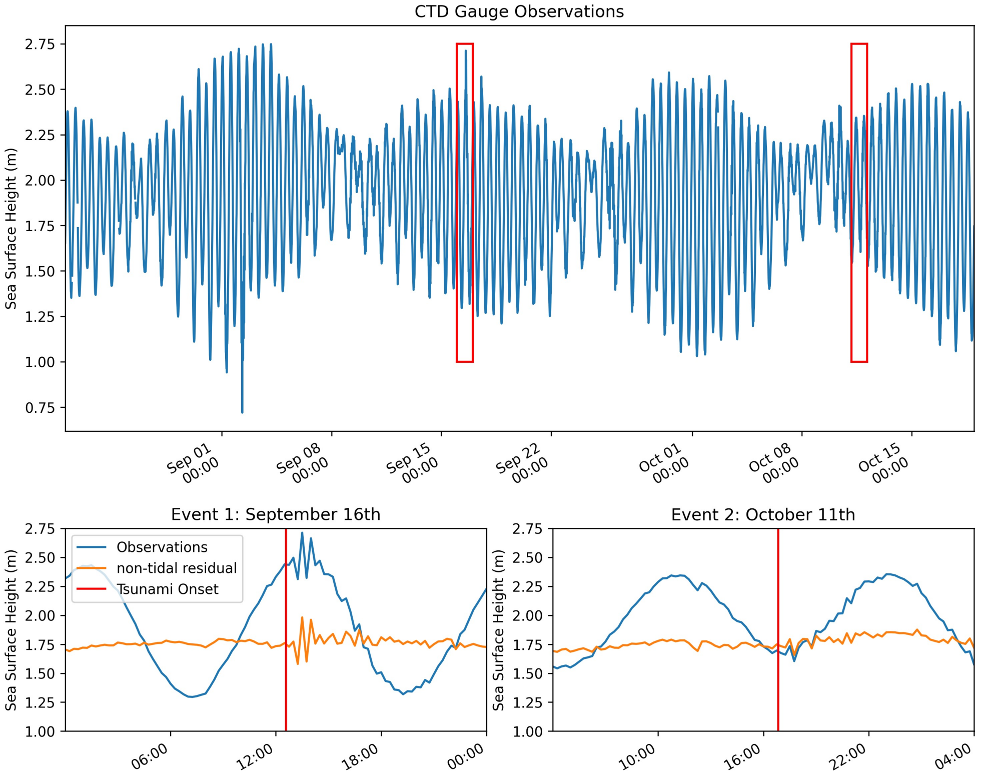

Supplementary Figure 5 Sea level observations from the CTD gauge located in the inner reaches of the Dickson fjord during the September and October events [3]. Observations and non-tidal residuals are shown for the hours before and after tsunami onset. Observations are sampled at 15 minute intervals.

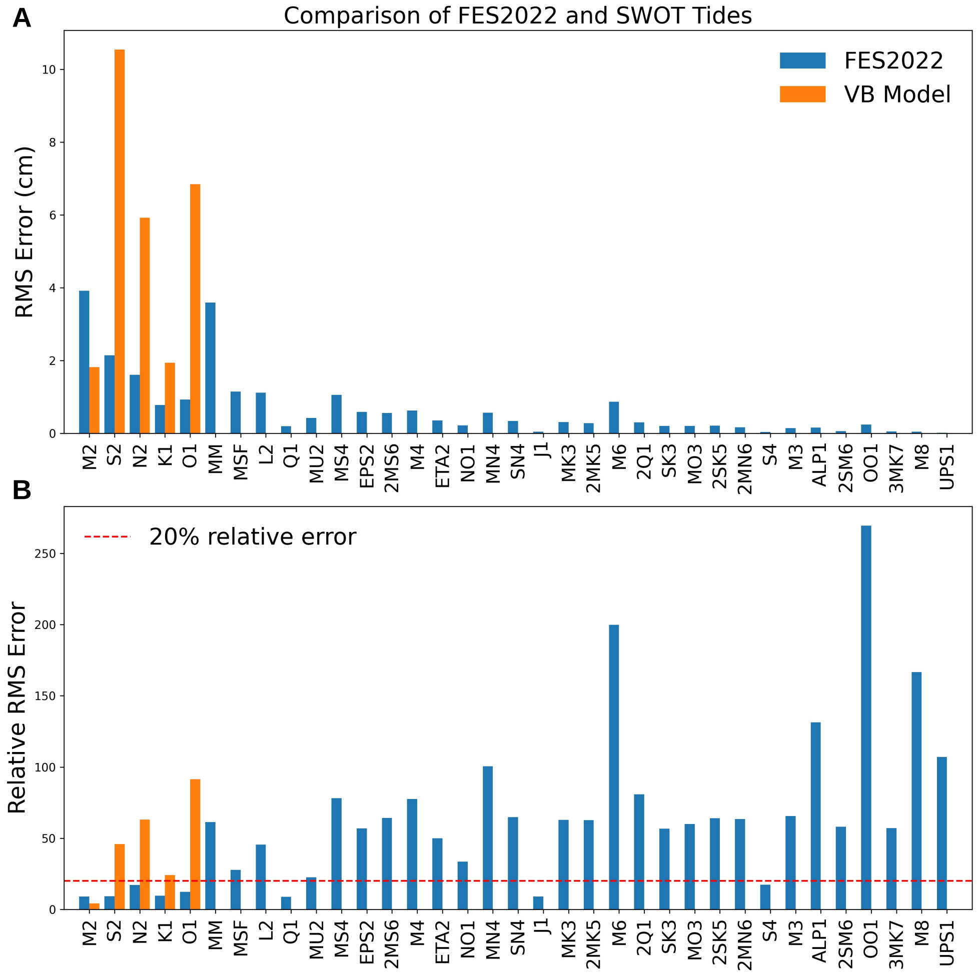

Supplementary Figure 6 Comparisons of FES2022 [4] and VB derived tides from SWOT with the in-situ CTD Gauge. Upper Panel shows the RMS error defined in Section 4.0.7. Lower Panel shows the relative RMS error to the magnitude of the CTD gauge constituent. Note VB tides are only estimated for M2, N2, S2, K1, and O1.

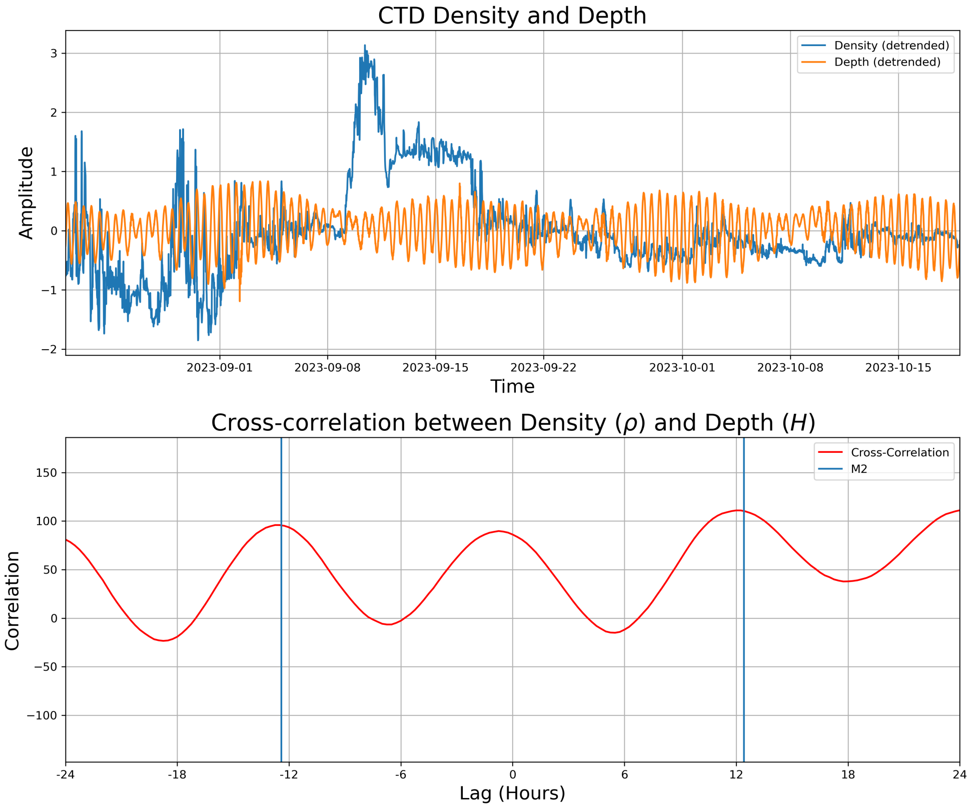

Supplementary Figure 7 Cross-Correlation between depth and density at Dickson fjord CTD Gauge. Top panel shows timeseries of detrended sea-surface height and density respectively. Lower panel shows cross-correlation.

References

[1] European Space Agency. Copernicus Global Digital Elevation Model (2024). URL https://doi.org/10.5069/G9028PQB. Distributed by OpenTopography. Accessed: 2024-10-10.

[2] Met Office. *Cartopy: a cartographic python library with a Matplotlib interface*. Exeter, Devon (2010 - 2015). URL http://scitools.org.uk/cartopy.

[3] Boone, W. *et al.* Greenland Integrated Observatory - CTD & Atmospheric Station Dickson Fjord - 2023. https://doi.org/10.14284/637 (2023).

[4] CNES. Fes2022 (Finite Element Solution) Ocean Tide (Version 2022) (2024). The FES2022 Tide product was funded by CNES, produced by LEGOS, NOVELTIS, and CLS, and made freely available by AVISO.
